# Supplementary material for: Are carbon emissions trading and green financial instruments synergistic? -Comprehensive quantitative research based on content analysis
Source: PLoS One. 2024 Mar 7;19(3):e0298601. doi: 10.1371/journal.pone.0298601 (PMC10919601; doi:10.1371/journal.pone.0298601)
Supplement: S1 Data — (ZIP) [file pone.0298601.s001.zip › Data/地级市Z_F工作报告.zip!/┴╔─■/╕o╦│╩╨/└┤╫╘╣1⁄2╓┌║┼í╛┬φ┐╦╩2╛▌═°í┐_2010.docx]

各位代表：

　　现在，我代表市人民政府向大会报告工作，请予审议，并请市政协委员和列席同志提出意见。

　　一、2009年政府工作回顾

　　2009年，是新世纪以来我市经济发展最为困难的一年。面对国际金融危机带来的巨大挑战，在市委的正确领导下，在市人大和市政协的监督支持下，全市人民齐心协力，奋勇拼搏，完成了市十四届人大二次会议确定的目标任务，形成了经济发展、民生改善、社会稳定、政通人和的良好态势。

预计实现地区生产总值724亿元，比上年增长13.6%；地方财政一般预算收入53.8亿元，增长19%；全社会固定资产投资533亿元，增长40%；社会消费品零售额338.1亿元，增长18.1%；实际利用外资3.1亿美元，增长133.7%；城市居民人均可支配收入13557元，增长9%；农民人均纯收入6264元，增长12.7%；万元生产总值能耗降低5.1%。经济增长保持了不低于振兴以来的平均水平，不低于东部地区的平均速度。

　　应对金融危机成效明显。面对年初工业企业开工不足、工业用电量急剧下滑等严峻形势，我们采取一系列有力措施，有效扭转了经济下滑态势。工业经济止跌回升，规模以上工业增加值由一季度增长0.1%提高到全年的14.7%，实现利润总额41亿元，增速位居全省第二位；个体户升级为企业1046户，为上年的2.1倍；新增规模以上工业企业376户、限额以上商业企业185户，分别为上年的2.8倍和1.2倍。完成高新技术产业增加值110亿元，为上年的1.2倍。重点项目建设加快，超亿元项目开工建设160个，总投资1306亿元，当年完成投资379.5亿元，分别为上年的1.8倍和1.4倍。房地产投资额和商品房销售额创历史新高，分别为上年的1.6倍和1.8倍。完成全口径税收155亿元，为上年的1.7倍；全地区新增贷款167.9亿元，为上年的7倍；实现土地出让收入50.3亿元，为上年的1.7倍；旅游总收入由全省第6位升至第4位。

　　对外开放实现历史性跨越。加大主题概念和产业集群招商力度，全年签订合同项目156个，总投资人民币782亿元、外资30亿美元，分别为上年的2.1倍和5.5倍。其中投资10亿元以上项目23个，总投资人民币483亿元、外资22.3亿美元，分别为上年的1.6倍和7.2倍。引进总部企业80户。加大向上争取力度，全年争取资金60亿元，为上年的1.4倍；争取建设用地指标760公顷，为上年的3.7倍。特别是经过艰辛不懈努力，我市争取到国家最后一批资源枯竭型城市转型试点市，连续四年累计将获得国家一般性转移支付资金10亿元以上；抚顺铝厂政策性破产得到国务院批准，是两年来国家批准的唯一一家政策性破产企业；经济开发区被确定为国家唯一的先进能源装备产业化基地。一些长期困扰抚顺发展的重大问题得到解决。

　 沈抚新城建设突飞猛进。全年落地项目165个，总投资555亿元；投资10亿元以上项目14个，总投资329亿元；当年完成投资200亿元，是近3年总和的1.4倍。其中，产业区落地项目113个，总投资260亿元；核心区落地项目33个，总投资202亿元；生态区落地项目19个，总投资93亿元。全年投入32.5亿元，加快推进以“两湖两带、三纵四横”等为重点的基础设施建设和土地收储，为招商引资和项目落地创造了条件。在不久的将来，一座现代化的新城将在沈抚连接带上崛起。

　　县区经济发展迅猛。明确了县域工业产业集群和农业产业化主导产业定位，强化县城建设，县域经济发展势头强劲。预计三县地区生产总值、固定资产投资和财政一般预算收入增幅分别高于全市平均水平8.9、9和16.2个百分点。战胜严重旱灾，粮食产量达到52万吨。现代特色农业建设步伐加快，建成一批设施农业小区、畜牧业标准化小区和农业标准示范小区，规模以上农业产业化龙头企业达90家，带动农民19.6万户。农村基础设施建设进一步完善，新建小型灌溉水源工程482处，病险水库除险加固5座，整治河道86公里，防治水土流失8667公顷。巩固林改成果，建设森林经营试验示范林1万公顷，开发林地经济2.9万公顷。加大扶贫开发力度，投入财政资金2664万元，减少低收入人口4.3万人。县城建设迈出新步伐，清原、新宾两县房地产施工面积51万平方米，为上年的1.4倍。明确了城区服务业和工业产业集群定位，加大招商引资力度，四个城区签约超亿元合同项目42个，总投资338亿元；全面启动危旧楼改造，拆迁68万平方米，规划施工216.6万平方米。高新区建设取得较大进展，累计完成基础设施建设投资4.9亿元；签约合同项目15个，总投资67.4亿元；落地开工项目6个，总投资12.7亿元。

　　城乡建设步伐加快。全省第一条城际铁路通车运营，长期困扰城市交通的16个平交道口成为历史，24公里沿线两侧环境明显改观。南杂木至草市高速公路建设加快推进，永陵至桓仁、南杂木至通化高速公路全面开工，沈抚同城化2号路和南环路东延建成，滨河北路开工建设，改造城市主次干道41条、“摸黑路”26条，改造供热主干管网23公里。投入财政资金8700万元，强化市政设施维修养护。更新公交车辆251台，公交IC卡售票系统正式运行。加强综合整治，拆除违法建筑2500余处，清理违章占道5500余处；查扣无证无牌“黑车”743台，销毁非法出租车135台。努力改善生态环境，推进河滩清淤和码头建设，浑河中部段具备通航条件；整治浑河干支流21.6公里，城区植树134万株，新增绿地118公顷；城区1784台1吨以下燃煤小锅炉全部拆除改造，南杂木、永陵等5座城镇污水处理厂建成启用，化学需氧量和二氧化硫减排提前实现“十一五”规划目标。

　　和谐抚顺建设取得新成效。坚持尽心竭力为群众办实事，让改革发展成果最大限度地惠及百姓。继续保持零就业家庭动态为零，全年新增实名制就业11.2万人，城镇登记失业率控制在5%以内。加强社会保障体系建设，强化社会保险扩面征缴，积极筹措和争取资金，确保了27.5万离退休人员基本养老金按时足额发放。城镇居民基本医疗保险覆盖率达80%，国有及集体困难企业10.1万退休人员参加了职工医疗保险，职工基本医疗住院费个人负担比例明显降低。新型农村合作医疗实现全覆盖，住院补偿比例达到45%以上。大幅提高城乡低保、低保边缘户和农村五保户的保障供养标准，累计发放保障资金3.1亿元。实施“蓓蕾生命”慈善救助工程，221名城乡低保家庭患儿得到救治。改善群众住房条件，建设棚改安置用房42.8万平方米，改造农村危房2157户，配建廉租房3100户，发放困难群众住房补贴3555户，租金核减3700户。改造旧有房屋供水设施6800户，解决了6100户城市居民吃水难和4.1万农民饮水安全问题，解决了815户城市居民煤气拖期开栓问题。全面落实信访稳控责任制，投入2.9亿元，解决重点信访案件1392件，信访总量下降30.6%。全市没有发生重大安全事故，生产安全事故下降50%以上。严厉打击各种犯罪，刑事案件和“两抢一盗”发案率创2000年以来新低，人民群众安全感明显增强。

社会事业全面进步。各级各类教育健康发展，教育教学质量不断提高；促进教育公平，城乡16万学生义务教育阶段学杂费全部免除；加快发展职业教育，建成公共实训基地10个，毕业生就业率达90%以上；整合教育资源，撤并中小学39所，建成农村九年一贯制寄宿学校10所，改造城区薄弱学校13所。开展群众性文化广场活动2200场次，新建农家书屋209家，“新宾满族剪纸”被列入联合国“人类非物质文化遗产代表作名录”。市第三医院改造竣工并交付使用，新建改造县乡村医疗机构128个、社区卫生服务中心12个，甲型H1N1流感得到有效防控。人口出生率控制在5.4‰以内，人口素质进一步提高。新建健身场所120处，举办全民健身活动赛事36项，抚顺籍运动员在全运会上取得4金、2银和3铜的好成绩。

　　文明城市创建、双拥模范城建设、民族、宗教、外事、侨务、审计、统计、工商、质监、药监、金融、出入境检验检疫、防震、海关、烟草、气象、档案、人防、粮食、物价、供销、邮政、通讯、电力、老龄、妇女儿童、残疾人等各项工作都取得了新的进展。

　　政府自身建设不断加强。自觉接受市人大及其常委会的法律监督和工作监督、市政协的民主监督，办理人大代表建议341件、政协提案486件，按时办复率100%，满意率99.5%。坚持市长办公会和国土规划委员会例会制度，推进行政决策科学化和民主化。强化依法行政，取消和停止收费项目88项，市本级行政审批时限缩短50%，政府还贷普通公路收费站全部撤销。实施机构改革，政府部门由43个调减为37个。强化作风建设，争分夺秒，夙兴夜寐，求发展、谋振兴的氛围已经形成。

　　各位代表，刚刚过去的一年，是我市遭受金融危机冲击异常严重、经济形势异常严峻、稳定任务异常艰巨的一年，是应对危机措施有力、经济逐步企稳回升、社会大局和谐稳定的一年。一年来，我们遇到的困难前所未有，承受的压力异常沉重，付出的努力超乎寻常，取得的成绩弥足珍贵。这些成绩的取得，是市委正确领导的结果，是市人大和市政协监督支持的结果，是抚顺石化公司、抚顺矿业集团等企业和各条战线共同努力的结果，是全市人民拼搏实干的结果。在此，我代表市人民政府，向全市人民致以崇高的敬意和诚挚的感谢。

　　总结去年的工作，我们也清醒地看到，我市经济社会发展中还面临不少困难和问题。一是历史包袱沉重，国企改革滞后，经济结构不尽合理，政府债务、城建欠帐、养老保险缺口和地质灾害治理等压力巨大，一些长期积累的深层次矛盾尚未根本解决。二是私搭乱建和私挖乱采时有发生，房屋拆迁、土地征用、环境保护、物业管理和冬季供暖等方面存在薄弱环节，一些涉及群众切身利益的问题还没有很好解决。三是一些部门办事效率不高、主动服务意识不强，推诿扯皮和吃拿卡要现象时有发生，政府自身建设还需进一步加强。对于这些矛盾和问题，我们必须下大力气，认真加以解决，决不辜负全市人民的期望。

二、2010年政府主要工作任务

　　今年是实施“十一五”规划的最后一年，也是抚顺实现大发展的关键一年，做好今年工作，意义重大。我们必须坚定信心，保持昂扬向上的精神状态，满怀发展激情，创造性开展工作。只要再接再厉、乘势而上，抚顺就一定会发展得更好、更快，抚顺人就一定能创造更加辉煌的未来。

　　今年政府工作的总体思路是：全面贯彻党的十七大和十七届三中、四中全会精神，深入落实科学发展观，紧紧抓住东北振兴和沈抚同城化的历史机遇，以招商引资上项目为中心，大力实施“两区两带”发展战略，举全市之力建设沈抚新城，突出发展壮大县区经济和产业集群两个重点，更加注重提高经济增长质量和效益，更加注重推动经济发展方式转变和经济结构调整，更加注重推进改革开放和自主创新，更加注重环境保护和生态文明建设，更加注重改善民生和促进社会和谐稳定，努力实现抚顺经济社会发展全面提速，全面完成“十一五”规划任务，为“十二五”实现新跨越奠定坚实基础。

　　今年全市经济社会发展的主要预期目标是：地区生产总值增长14%，财政一般预算收入增长20%，全社会固定资产投资增长40%，实际利用外资增长29%，城市居民人均可支配收入增长12%，农民人均纯收入增长12%，万元生产总值能耗降低5%。力争地区生产总值、固定资产投资突破“双八百”，力争一些主要经济指标增速走在全省前列。

　　实现上述目标任务，我们重点抓好以下九个方面工作。

　　(一)大力实施“两区两带”战略，为3-5年再造一个抚顺奠定基础

　　建设“两区两带”，是市委、市政府站在抚顺全面振兴高度作出的重大决策，是全市今后的工作重点和主要任务，必须一以贯之，一抓到底。

　　加快沈抚新城建设。举全市之力，集各种资源，确保沈抚新城三年基本形成规模。今年主要经济指标必保增长50%以上，签约合同项目150个，总投资300亿元。核心区要重点打造景观节点，完成两湖水系和浑河公园建设，积极推进总投资300亿元的总部、商业和地产项目建设。产业区要重点拓展发展空间，完成10平方公里土地收储和基础设施建设，落地开工项目100个，完成投资200亿元。生态区要加快“退二进三”步伐，积极推进总投资200亿元的旅游和地产项目建设，加快推进325库搬迁和综合开发，启动方晓新城建设。拓宽改造沈抚大道和高望线，形成与沈阳相连的12条通道。

　　加快高新区建设。围绕石化资源特别是“百万吨乙烯”产品的承接深加工，加大招商引资力度，全年新签项目合同额100亿元，实际到位资金20亿元。强化基础设施建设，强力推进项目落地，4平方公里工业园区的项目落地率达70%以上。确保高新区列入省级高新技术产业开发区。

　　加快南环产业带建设。完成产业发展规划，积极筹建南环铁路，打造新的经济增长带。重点发展循环经济产业集群，全力推进抚矿集团总投资270亿元的200万吨再生造纸、2×30万千瓦煤矸石热电机组、页岩油深加工示范等重点项目建设。

　　加快浑河景观带建设。以“沈抚黄金带、水岸新城区”为主题，统一规划，成片开发，分步实施，用三年左右的时间，将浑河两岸打造成为生态商住景观带和城市地标带，形成展示抚顺新形象的靓丽风景线。今年要全面启动“一河、两线、四区”建设。一河，即综合整治百里浑河城市段干支流。两线，即规划设计改造滨河南路，全线贯通滨河北路。四区，即启动总面积68平方公里的东部东湖地区、中部城市段、月牙岛地区和西部沈抚新城的浑河两岸规划建设。

（二）坚持改革开放，增强地区发展活力和后劲

　　加大国企改革力度。稳妥推进抚顺铝厂、抚顺特钢等8户企业政策性破产，加快60户进入程序的国企改制，逐步解决138户已改制企业遗留问题。稳步推进供销社系统企业改革。

　　加大对外开放力度。坚持招商与选商相结合，进一步提高招商项目质量。突出主题概念和产业集群招商，重点面向资本密集区、行业龙头企业、中央大企业和世界500强，开展点对点、面对面招商。全年引进超千万美元的外资项目15个、超亿元的内资项目80个，实现域外引资到位额406亿元，增长40%。鼓励企业扩大出口，支持有条件的企业境外投资。

　　加大项目建设力度。坚持域外、域内项目并重，全面落实重大项目协调推进机制，强化重大项目考核和责任追究制，及时解决立项、环评、安评、土地、规划等问题，确保签约项目早落地，开工项目快建设，力争总投资2550亿元的268个超亿元重点项目完成投资752亿元，其中60个投资10亿元以上项目完成投资461亿元。

加大向上争取力度。及时把握国家政策，研究申报一批符合国家产业目录和资金投向的项目，争取国家和省政策、资金支持取得新进展，争取国家将我市确定为首家矿山地质灾害综合治理试点市。

（三）以增量调整工业结构，提高经济增长质量和效益

　　做强优势工业。积极推进抚顺石化公司“千万吨炼油、百万吨乙烯”、抚矿集团转型项目、特钢集团技改工程3个投资超百亿元的工业项目。加快传统产业改造升级和高新技术产业发展，重点打造三大千亿产业基地，即沈抚新城千亿产值的先进装备制造业基地，高新区以碳纤维为重点的千亿产值的新材料、精细化工产业基地，南环产业带千亿产值的循环经济产业基地。大力发展战略性新兴产业，制定新兴产业工业化发展规划，推进新兴产业项目建设，努力培育新的经济增长点。

　 壮大地方企业。鼓励企业兼并重组,大力发展中小企业，促进非公有制经济发展。重点培育销售收入超亿元的工业企业100户、超10亿元的企业20户，新增规模以上工业企业200户、限额以上商业企业200户；重点培育产值超亿元的建筑业企业50户、超10亿元的企业5户,全年完成建筑业增加值50亿元，增长30%。

　　（四）围绕三年翻番目标，加快发展县区经济

　　大力发展县域经济。突出抓好县域工业园区建设，尽快形成有特色、有规模的工业产业集群。抚顺县加快拉古、兰山工业园区建设，大力发展工程机械零部件和精细化工产业集群；清原县加快清原镇工业园区建设，大力发展输变电配件产业集群；新宾县加快南杂木工业园区建设，大力发展林木加工产业集群。壮大农业产业化主导产业，尽快形成“一县一业”特色产业格局。抚顺县重点打造以单片黑木耳为主的食用菌产业，清原县重点打造以林下参和龙胆草为主的中药材产业，新宾县重点打造以香菇为主的食用菌产业。科学规划建设县城，重点推进新宾县、清原县两座星级酒店建设和地产开发，重点支持以兰山小城镇为代表的新农村建设。加快发展现代农业，新建设施农业667公顷、畜禽标准化养殖小区178个、标准化生产示范基地10个，开发林地经济2.7万公顷。加强农业基础设施建设，中小型水库除险加固12座，建设小型农田水利工程438项，改造中低产田3000公顷，确保农村生产生活条件改善和粮食增产丰收。

大力发展城区经济。依托优势，体现特色，错位发展，做大做强产业集群。新抚区围绕打造中心商贸区，大力发展商贸集群、市场集群和仓储物流集群，力争进入全省服务业十强区。顺城区立足前水后山的自然条件和现有企业的优势，重点发展起重设备产业集群，打造宜居商住新区。东洲区依托“千万吨炼油、百万吨乙烯”和萨尔浒风景区等资源优势，打造精细化工产业集群，大力发展休闲旅游和房地产业。望花区依托大企业和科研院校集中的优势，大力发展冶金和造纸产业集群。

（五）坚持规模与特色并重，推进服务业大发展

　　加快发展商贸业。重点推进总投资253亿元的旺力国际商城、银江科技城、香港综合娱乐城、商氏综合娱乐城、欧亚春城、圣大国际商贸城、天朗现代城、舜泰三悦城、东方新天地、世城财富广场和站前地下商城等11个单体投资超10亿元的服务业项目建设，加快推进新抚、顺城、望花和开发区5个五星级酒店建设。加快构建新型市场体系，积极推进华山物流园区建设，抓好榆林专业和仓储物流等一批市场建设，组织好家电、汽车、摩托车下乡和“万村千乡”活动。实现社会消费品零售额400亿元，增长18.4%。

　　加快发展旅游业。做强做优红河峡谷漂流、皇家极地海洋世界、三块石、清永陵和元帅林等特色品牌旅游项目，重点推进总投资80亿元的丰远?热高乐园、聚隆滑雪场、金山石佛、大伙房水库坝下旅游综合开发、青龙寺和猴石森林公园等旅游项目建设，丰富地区旅游资源。加大国内外旅游招商引资力度，力争引进一批大型旅游项目，用三年时间将抚顺打造成为旅游强市。

　　加快发展房地产业。促进工业地产、商业地产和住宅地产全面发展，进一步活跃房地产业。结合城区“退二进三”，大规模实施旧城区改造。加强对房地产开发企业的服务和管理，促进房地产业健康发展。全年完成房地产开发投资77亿元、增长20%，实现商品房销售额58亿元、增长18%。

　　加快发展金融业。支持金融企业改善资产质量，促进金融企业发展。加强银企对接，鼓励金融企业扩大信贷规模，力争存贷比达到全省平均水平。鼓励企业进入资本市场，年内实现1户企业主板上市，做好5户企业上市准备。完善金融服务体系，组建村镇银行、小额贷款公司和担保公司21家。大力发展总部经济，鼓励国内外知名企业在抚设立区域总部、研发中心、销售公司和商务代表处。

（六）科学规划建设，全面改善城市环境

　　提高规划水平和控详规划覆盖率。坚持“先规划、后建设”，加快编制沈抚新城核心区、浑河景观带等重点区域的控详规划，使每块土地都列入规划，每个项目都按规划实施。以中心城区、浑河两岸等地段为重点，搞好城市空间形态、建筑风格与色彩设计。严格规划审批和监管，坚决维护规划的严肃性和权威性。

　　加强基础设施建设。重点推进抚草、永桓和抚通3条高速公路建设，抚草高速公路年末竣工通车；实施中央大街北延工程，使中央大街与浑河南路相连；建设东环大道，形成城市东部南北大通道；实施南环路西延工程，与沈阳十大线相连；完成滨河南路快速干道东延至大伙房水库坝前工程，打造景观路；规划贯通绥化路、青年路、鞍山路和洗化东路，形成城区东西新干道。加大“三供”管网改造力度，启动输水暗灌改造工程，继续改造望花、刘山、古城子地区排水设施，建设大伙房输水二期工程抚顺西出口配套项目，新建城市卫生填埋垃圾场。

　　强化城市管理。完善市、区、街道和社区四级管理体制和联动机制，强化综合执法，充分发挥城区政府管理职能。坚持集中整治与长效管理相结合，突出整治脏乱差、违章建筑和非法营运。加强对各类住宅小区的物业管理，提高居民满意率。积极协调解决东岗道桥收费问题。

启动生态城市建设。继续整治望花等重点区域大气污染，逐步拆除20吨以下燃煤锅炉，力争5年内城区全部实现集中供热，全年化学需氧量和二氧化硫排放量分别控制在3.3万吨和7.9万吨以内。全面实施大伙房水库周边及上游环境综合整治，大伙房水库保持二类水质。改造新建污水处理厂4座、垃圾渗漏液处理厂1座，综合治理演武等垃圾场。环城林带造林补植600公顷，城区植树100万株，新增绿地100公顷。全面实行封山禁牧，保护青山绿水。

（七）统筹兼顾，协调发展各项社会事业

　　优先发展教育事业。以提高教育教学质量为核心，整合教育资源，优化教育布局，推进素质教育，强化师德建设，建立教师规范化管理长效机制，促进义务教育均衡发展。建立健全基础教育、职业教育和高等教育协调发展的教育体系，最大限度地满足社会多样化的教育需求。新建农村九年一贯制寄宿学校2所，完善农村九年一贯制寄宿学校附属设施10所，中小学校舍安全工程完成三年规划目标的60%，“双高普九”达标率达到60%以上。积极推进抚顺职业技术学院与抚顺师专的整合搬迁。

　　大力发展文化卫生体育事业。积极开展群众性文化活动，繁荣文化事业，壮大文化产业，全面实施文化信息资源共享工程，重点推进抚顺大剧院、群众艺术馆、科技馆、青少年宫、档案馆和13座乡镇文化站建设，加强网吧监管整治，完成第三次全国文物普查。深化医药卫生体制改革，加快基本公共卫生服务均等化，新建县乡村医疗机构112所，改造社区卫生服务中心15所，搬迁新建市中医院，推进第四医院、第二医院改扩建。广泛开展全民健身活动，加快发展体育产业，新建健身场所80处，启动市体育馆改造和网羽球馆建设，做好省运会的参赛工作。继续稳定低生育水平，实施免费婚检，提高出生人口素质。

　　搞好第六次人口普查，做好民族宗教、外事侨务、社会福利、妇女儿童、残疾老龄、优抚安置、气象防震、减灾救灾、国家安全和双拥共建等各项工作。

(八)坚持以人为本，促进社会和谐稳定

　　继续扩大就业。坚持以创业带动就业，多渠道扩大就业，全年普惠制就业培训3.9万人，城镇实名制就业9.5万人，城镇登记失业率控制在5%以内，零就业家庭动态为零。

　　完善社会保障体系。以个体劳动者为重点，强化社会保险扩面征缴，确保企业离退休人员基本养老金的按时调增和足额发放。积极推进清原县新型农村养老保险试点。继续做好城镇居民和困难企业退休人员医疗保险工作。巩固新农合成果，进一步提高新型农村合作医疗参保补助标准。强化低保对象动态管理，适时提高城乡低保户和五保户供养标准。深入开展扶困助学、精神康复和助孤抚老等慈善活动。

　　改善群众生活条件。新建改造农村危房700户，解决农民吃水难3万人。以租金核减、发放经济适用住房和租赁住房补贴为重点，加大住房保障力度，拆迁改造零散棚户区24万平方米、危旧楼51万平方米。改造城市“摸黑路”35条、老旧房屋室内供水设施1万户。

　　建设平安抚顺。完善社会治安防控体系，严厉打击各种犯罪，不断增强人民群众安全感。加强群众来信来访工作，妥善处理社会矛盾，切实解决群众关心的热点难点问题。加强安全生产，保障食品药品和公共安全，坚决遏制重特大事故发生。

　　推进信用抚顺建设。弘扬雷锋精神，加强文明城市建设，努力创建和谐社区。以政府信用、企业信用和个人信用建设为重点，加强社会信用体系建设，形成诚实守信的社会道德风尚。

（九）加强自身建设，打造人民满意政府

要勇于创新。坚持把思想解放贯穿于各项工作始终，用创新的思维、创新的思路、创新的举措，解决影响改革发展稳定中的问题。要保持一股知难而上的勇气，一股敢为人先的锐气，一股干不上去誓不罢休的志气，把全部心思和精力用在谋发展、促振兴上，做出经得起人民、历史和实践检验的工作业绩。

要务实高效。坚持说实话、办实事、求实效，提倡开短会、发短文、讲短话，多做打基础、利长远、惠民生的工作，坚决反对官僚主义和形式主义。强化目标责任、强化督查督办、强化绩效考评，建立完善“零障碍、高效率”和“急事快办、特事特办”的政务绿色通道。

要依法行政。自觉接受市人大及其常委会的法律监督、工作监督和市政协的民主监督，坚持重大事项向人大报告、向政协通报制度，认真落实人大及其常委会的决议、决定，积极支持政协履行政治协商、参政议政职能，认真办好人大代表建议和政协提案。强化政务公开，提高政府工作透明度，增强人民群众的知情权、参与权和监督权。

要勤政廉洁。坚持从严治政，强化权力监管，加大对工程建设、土地出让、政府采购、工程招投标、国有资产处置和大额资金使用等重点领域的行政监察和审计监督力度，坚决纠正损害人民群众利益的不正之风，严肃查处违纪违法案件，努力建设一支干净、干事的公务员队伍，树立政府为民、务实、勤政、清廉的良好形象。

　　各位代表，时代赋予重托，拼搏铸就辉煌。重任在肩，我们还须奋力前行；前程锦绣，我们更要风雨兼程。让我们在市委的坚强领导下，团结带领全市人民，坚定信心，务实苦干，以更顽强的斗志、更务实的作风、更有力的措施，加快推进抚顺的科学发展、和谐发展、全面发展，为抚顺老工业基地重振雄风，为全市人民生活更加美好而努力奋斗！
